# Increased Risk of Esophageal Cancers Among Men in Taiwan

**DOI:** 10.7759/cureus.6990

**Published:** 2020-02-14

**Authors:** Olivia Dix, Mala Thakur, Alessia Genova

**Affiliations:** 1 Internal Medicine, Xavier University School of Medicine, Oranjestad, ABW

**Keywords:** esophageal cancer, taiwan

## Abstract

Despite increased research, the risks of developing esophageal cancer and of death from this disease, especially among men, have increased worldwide. The goals of this review include an assessment of male predominance of esophageal cancer and an analysis of its high mortality rates. Determination of the genetic and environmental factors associated with esophageal cancer may help explain its male predominance and help design more effective treatments of this disease.

## Introduction and background

Esophageal cancer is the eighth most common form of cancer and the sixth most frequent common cause of cancer deaths worldwide. The five-year survival rate among patients with esophageal cancer is 19%, decreasing to 0.9% in patients with advanced esophageal cancer [1]. Most patients are initially diagnosed with advanced-stage disease, with fewer diagnosed early enough for successful treatment. Esophageal cancer may be due in part to the lifetime exposure of the esophagus to many environmental factors, including irritants, spices, hot food and drink, alcohol, and different types of smoke. 

The prevention and treatment of esophageal cancer are difficult due to the location of the esophagus, an organ hidden within the body by other vital organs-lack of access blocks both the diagnosis and surgical removal of esophageal tumors [2]. Esophagectomy is regarded as the initial approach to manage esophageal cancers, especially in their earlier stages, whereas endoscopic procedures, such as endoscopic mucosal resection, are used to remove advanced-stage tumors. The latter tumors require more extensive management, including chemotherapy and/or radiotherapy. Tumor removal may not be effective, as many patients experience postoperative complications [3]. The high mortality rates in these patients may be due to the methods used to manage this disease or the disease itself [4]. 

The incidence of esophageal cancer, especially of primary esophageal adenocarcinoma (EADC), has increased over the past few decades in both developed and non-developed countries. This increase is particularly pronounced in men, with its incidence over the last decade increasing from 0.09 to 0.75 per 100,000 persons per year. In non-developed parts of Taiwan, the male-to-female prevalence of esophageal cancer is approximately 9:1. The increased overall prevalence of esophageal cancer has been ascribed to poor nutrition, exposure to irritants such as alcohol and cigarette smoke, and increased age of the population [5]. 

Esophageal cancer is caused primarily by extrinsic factors and, to a lesser extent, by genetic factors. The increasing incidence of this disease in Taiwan and other countries may be due to poor lifestyle choices which could potentially be the reason of a continuous growth in tumors. Moreover, the availability of fresh produce, clean water, and clean air is lower in developing than in developed countries. People living in low-income areas of Taiwan consume more alcohol and tobacco products than those living in higher-income areas, increasing the risk of malignant tumors. In Taiwan, a higher percentage of men than women smoke cigarettes or use other tobacco products that contain carcinogens, thus explaining, at least in part, the higher incidence of esophageal cancer in men [6].

The esophagus is exposed to the external environment, including various chemicals and temperatures, through breathing, eating, and swallowing. These chemicals and temperatures may disrupt homeostasis within the cells of the esophagus. The body, however, may be unable to detect an imbalance in homeostasis within these cells, with fewer symptoms occurring in patients diagnosed with esophageal cancer. During later stages of the disease, patients may experience obstruction or dysphagia, but successful or curative treatment may be impossible at these stages. Complete surgical resection may be too difficult, and chemotherapy and radiotherapy may be insufficient after tumors have metastasized [7]. Research is needed to identify curative treatments for patients with esophageal squamous cell carcinoma (ESSC) and EADC.

## Review

Several primary studies have explored the relationship between genetic factors and male dominance of esophageal cancer in Taiwanese patients. One of the sulfotransferase group 1A enzymes, SULT1A1, is a key enzyme involved in the metabolism of drugs, neurotransmitters, hormones, and xenobiotics that act as toxins within the body [8]. Male patients from three medical centers in Taiwan diagnosed with ESSC between 1996 and 2001 were genotyped by polymerase chain reaction to identify SULT1A1 alleles within this population. Three alleles were identified: arginine/arginine, arginine/histidine, and histidine/histidine. The risk of developing esophageal cancer was higher in Taiwanese men with the arginine/histidine allele than in those with the two homozygous alleles. In addition to showing that genetics are involved in the development of esophageal cancer, these findings suggest that detection of the arginine/histidine allele may be a marker for early-stage esophageal cancer. 

Sex hormones may also be associated with the development of esophageal cancer. One study measured circulating concentrations of the sex hormones estrogen, progesterone, testosterone, and luteinizing hormone (LH) in men and women to assess the relationship between esophageal cancer and gender differences [9]. The female hormone estrogen plays a major role in menstruation, fertilization, and implantation and remains at high levels throughout a woman’s menstrual cycle, whereas another female hormone progesterone maintains the lining of the uterus for implantation of fertilized eggs [10,11]. The male hormone testosterone regulates fertility, enhances muscle mass, and is involved in distribution [12]. Although LH is present in both males and females, it mainly functions in men to increase sperm and testosterone production [13]. This Taiwanese study suggested that, due to reproductive factors, the risk of EADC was lower in women than in men [9]. The concentrations of circulating sex hormones vary greatly throughout a woman’s lifetime [13]. Menarche reduces esophageal cancer development by increasing estrogen and progesterone concentrations. Breastfeeding may also reduce the risk of EADC by increasing the levels of sex hormones, such as estrogen, oxytocin, and progesterone. A large study in the UK among 1.3 million women found that their risks of developing esophageal cancer had decreased, providing further evidence for the correlation between female sex hormones and the risk of EADC [9].

The concentrations of male sex hormones, such as testosterone and LH, were found to be higher in men who were diagnosed with esophageal cancer than those who were not, providing further evidence for the higher risk of esophageal cancer in men than in women [9]. The concentrations of these hormones may be diagnostic for early-stage esophageal cancer in men. 

Barrett’s esophagus is highly prevalent in patients with esophageal adenocarcinoma due to changes in cell growth within the esophagus. Men were reported more likely to develop types of diseases that affect the cells in the esophagus, making them more prone to develop cancerous cells. The magnitude and prevalence of erosive reflux disease are higher in men, resulting in a higher male prevalence of damaged cells in the esophagus due to the acidity of stomach contents traveling to the esophagus. The resultant distortion of homeostasis within the esophagus could lead to more damage and potential abnormalities within esophageal cells. Although there was no correlation between reflux disease and the male predominance of esophageal cancer, further studies are required [9]. More research on patients with Barrett’s esophagus may result in a greater ability to predict prognosis in patients with esophageal cancer.

Environmental factors are associated with an increased risk of developing esophageal cancer in Taiwan. The most common substances used worldwide are caffeine, tobacco, and alcohol [14]. In Taiwan, however, other factors are more common. For example, many Taiwanese people consume betel quid with or without tobacco, and many chew areca nuts combined with tobacco. Betel quid is a nervous system stimulant that increases circulating concentrations of adrenaline and noradrenaline [14]. Betel quid, a stimulant similar to tobacco, contains alkaloids that have pharmacological effects on both the sympathetic and parasympathetic nervous systems, increasing body temperature and pulse rate, improving concentration, heightening alertness, staving off hunger, and lifting one’s mood [14]. 

The consumption of betel quid in Taiwan was found to increase fivefold in 15 years, with the consumption of betel quid with tobacco being much higher in the Taiwanese than in the Indian population [14]. In the latter, both betel quid chewing and alcohol drinking were the two highest risk factors for developing esophageal cancer [15]. In Taiwan, males were found more likely than females to smoke betel quid due to their type of work, and about 20% of male bluecollar workers in Taiwan being betel quid chewers. Cultural factors influencing male smoking include smoking by their fathers. The incidence of cancer deaths due to betel quid consumption increased by 60% from 1996 to 2002 [14]. The study was somewhat inclusive as most people in Taiwan who chewed betel quid were also heavy cigarette smokers. However, betel quid is classified as a group 1 carcinogen based on its increased risk of cancer in the oral cavity, pharynx, and esophagus. Taiwanese men are more likely to smoke betel quid with tobacco and cigarettes than Taiwanese women, both of which contain risk factors for esophageal cancer. This may explain the prevalence and male predominance of esophageal cancer in Taiwan. Overexposure of esophageal cells to these carcinogens may induce mutations and abnormal cell growth [2]. 

Alcohol consumption is another risk factor for esophageal cancer. One study reported that alcohol and tobacco were major risk factors for esophageal cancer in central and southeastern Asia, including Taiwan [9]. Because the esophagus is in direct contact with alcohol, alcohol has a greater effect on the esophagus than on other organs. Because men are more likely to drink alcohol and smoke cigarettes than women, men are more likely to develop esophageal cancer [14]. A study assessing the correlation between alcohol drinking and the development of EADC in Taiwanese women found that ethanol could potentially affect the esophageal mucosa and that alcohol-associated toxicity or oxidative damage could penetrate the mucosal layer, causing damage to that layer. Greater damage to this layer could lead to damage to specific areas of the esophagus by active carcinogens, resulting in malignancies [16]. 

Overall, studies have suggested hypotheses for the greater risk of esophageal cancer in men than in women. Other factors may also be involved, including the high costs of healthcare ihn Taiwan and the relatively poor equipment, resulting in a lack of accurate diagnoses. Although the high out-of-pocket fees paid by patients may prevent them from visiting doctors, Taiwanese individuals tend to seek medical help even for minor ailments. The resulting high patient-doctor ratios may result in hospital overcrowding, reducing the quality of care for individual patients. Cancer diagnosis and treatment requires many tests and other expensive procedures, as well as potent drugs, which may be costly in Taiwan. Currently, the Taiwanese national healthcare system does not take in enough money for premiums to cover the full costs of hospital visits [17]. 

Esophageal cancer is a major healthcare problem in Taiwan, with a higher prevalence in men than in women. Both genetics and environmental factors contribute to the development of esophageal cancer. Individuals with the STAT1A1 arginine/histidine allele are at greater risk factor for esophageal cancer than those with the arginine/arginine and histidine/histidine alleles, with men in Taiwan being more likely to carry the arginine/histidine allele than women [8]. Sex hormones may also be an important aspect of male predominance because of the fluctuation of female hormones. Other diseases, including reflux diseases, which are more common in men, may also be risk factors for esophageal cancer.

Environmental risks are also important. Men and women who developed EDAC were also exposed to cigarette smoke, alcohol, and/or betel quid. Taiwanese men are more likely to consume betel quid and smoke cigarettes than Taiwanese women, suggesting a correlation between these risk factors and esophageal cancer deaths in Taiwanese men. Similar gender differences have been observed in other populations throughout the world. 

## Conclusions

Factors associated with the development of and deaths from esophageal cancer have not been fully determined. Research of methods to monitor the development of esophageal cancer is continuing, especially in patients with Barrett’s esophagus. Despite improvements in technology, mortality rates from esophageal cancer are still increasing. Genetics and lifestyle may play roles in the natural history of esophageal cancer. Early diagnosis of this disease is necessary to reduced mortality rates.
